# The Impact of Joining a Team on the Initial Trust in Online Physicians

**DOI:** 10.3390/healthcare8010033

**Published:** 2020-02-06

**Authors:** Jia Li, Xinyu Bao, Xuan Liu, Ling Ma

**Affiliations:** School of Business, East China University of Science and Technology, Shanghai 200237, China; jiali@ecust.edu.cn (J.L.); y30181304@mail.ecust.edu.cn (X.B.); maling@ecust.edu.cn (L.M.)

**Keywords:** e-health, e-consultation, physician trust, brand extension theory, team strength, team similarity, team size, initial trust problem

## Abstract

*Introduction:* Trust is a major challenge for the online market and this is especially the case for e-consultation platforms. Research that promotes online physician trust is highly desirable. In this study, we focus on whether joining a team led by a well-known physician will increase physician trust and what team characteristics will affect this trust. *Materials and Methods:* Brand extension theory is applied to the healthcare context to explain the impact of joining a team on physician trust. Specifically, both team strength and team similarity are hypothesized to have the main effects. In addition, team size is hypothesized to have a moderating effect. A 2 × 2 × 2 experiment was conducted to test the proposed research model. *Results:* The results indicated that joining a team would significantly increase physician trust (*p* < 0.001). Both team strength (*p* < 0.001) and team similarity (*p* < 0.001) had positive impacts on physician trust. In addition, a larger team size resulted in a reduced positive effect of team strength on physician trust (*p* < 0.001). *Conclusions:* Joining a physician team is an effective and low-cost method to address the initial trust problem of unknown online physicians.

## 1. Introduction

A major challenge in the online market is building user trust [1,2], because many face-to-face clues are missing in virtual cyberspace and buyers usually have little or no prior interaction with the sellers. As a result, establishing trust in online sellers can be even more difficult than in the offline context. However, trust is very important for the online market. Without trust, users are less willing to participate in online transactions, hindering market prosperity. Therefore, many mechanisms have been proposed, such as third party payment (Escrow Services) [2] or the reputation system (online customer review) [3]. The effects of these mechanisms on trust improvement have been validated by many e-commerce research studies [2,3].

E-consultation is a special type of online market that focuses on delivering healthcare services online [4]. This new type of online healthcare consultation can reduce both waiting time and travel expenses [5]. It is also likely to be a valuable option in terms of providing patients with more efficient diagnoses. Trust is even more important for the e-consultation market, because the choice of a healthcare provider is a serious decision and the healthcare market has high information asymmetry [6]. Due to the “Superstar” or the “winner-take-all” effect [4], the online healthcare market is more concentrated than the offline one. Although well-known physicians earn trust online more easily, unknown physicians experience more difficulty gaining user trust. Consequently, a few very good doctors will be extremely busy, but some other unknown doctors will be idle [7]. In addition, unknown physicians experience an initial trust problem. The initial trust problem applies to unknown physicians who do not have enough initial trust, so they will not have any ratings, followers, or appointments. However, if a physician does not have any ratings, followers or appointments, she is less likely to gain user trust [8]. Therefore, how to build initial trust in unknown physicians is an important research question. 

The physician team is a new feature in many e-consultation websites such as guahao.com, a leading e-health platform in China. The physician team consists of members with similar skills or backgrounds. The physicians in the team collaborate with each other to deliver healthcare. The team usually has a well-known physician as the leader. The team size can either be small (approximately 5) or large (25+). A physician can decide whether to join a physician team and which team to join. The physician team leader will decide whether to accept a request to join the team. By joining the team, the physician might receive some benefits of perceived trust because the team might provide a quality endorsement, so trust is transferred from the team members to the focal physician. Although the online physician teams on e-consultation platforms have received a lot of attention recently [9,10], the effect of joining a physician team on perceived trust has not been tested. In addition, we do not know what team characteristics will influence the trust gained. Therefore, in this study, we focus on whether joining a team led by a well-known physician will increase physician trust and what team characteristics will impact trust in a physician.

The importance of physician collaboration and the positive effects of physician teamwork have been demonstrated by prior studies. The benefit of teamwork might include shared responsibility, reduced errors, reduced stress, and quicker recovery. Recent evidence suggests that improvements in teamwork in healthcare can lead to significant gains in patient safety, measured against the efficiency of care, complication rates, and mortality [11]. For example, Baggs et al. [12] investigated the association between the collaboration of intensive care unit (ICU) physicians and nurses and patient outcomes. The results indicate that medical ICU nurses’ reports of collaboration were positively associated with patient outcomes. Rafferty [13] explored the relationship between interdisciplinary teamwork and nurse autonomy and its effect on patient and nurse outcomes and nurse-assessed quality of care. They found that higher teamwork scores were associated with higher levels of nurse-assessed quality of care, perceived quality improvement over the last year, and confidence among patients to manage their care when discharged. Nurses with higher teamwork scores also exhibited higher levels of autonomy and were more involved in decision-making. For example, Farland et al. [14] evaluated the effect of a pharmacist–physician collaboration on the achievement of diabetes-related control measures. They found that pharmacist–physician collaborative management at multiple practice locations and the type of setting had positive impacts on glycemic control and diabetes-related health maintenance. However, most of these research studies focused on the collaboration among different types of caregivers (e.g., between physicians and nurses or between physicians and pharmacists). In-depth research that explores the collaboration among physicians is limited, especially in the context of online-based services. One exception is a recent study by Liu et al. [9]. They considered four kinds of team diversity, including status capital diversity, decision capital diversity, online reputation diversity, and professional knowledge diversity, and investigated how team composition from the diversity perspective affects online doctor team performance and how leader reputation moderates the effect of team diversity on team performance.

Trust is another important topic in healthcare that has received a lot of attention from researchers. A patient’s trust in a physician is crucial for desirable treatment outcomes, such as satisfaction and adherence. A trusting relationship between a patient and a physician results in facilitated communication and medical decision-making, decreased patient fear, and better treatment adherence [15]. For example, Lee et al. [16] found that trust in physicians is associated with both self-rated health and therapeutic response. Hinnen et al. [17] found that lower levels of trust in one’s physician is associated with more distress over time, in more anxiety-prone individuals with cancer. Other studies have focused on the antecedents of trust and these factors included communication skills [18], emotional intelligence [19], attachment style [20], racial differences [21], and payment method [22]. For example, Fiscella et al. [18] found that patient-centered communication is associated with increased trust. Weng [19] demonstrated that higher nurse-rated emotional intelligence scores of physicians had positive effects on patient trust. However, few studies have investigated the impact of team organization on physician trust, especially the effect of joining a group on a physician’s perceived trust.

As the major motivation for the unknown physician to join a team is to obtain a quality endorsement from a well-known team leader and other team members, we used the brand extension theory as the theoretical perspective in this study. We are especially interested in the actual effect of joining a team on physician trust. Therefore, the first research question of this study can be interpreted as follows:

RQ1: Will joining a team increase physician trust?

When a physician decides to join a team, she must decide which team she should join. In other words, we are interested in understanding how team characteristics will influence the physician’s trust. Therefore, the second research question of this study can be interpreted as follows:

RQ2: What team characteristics will impact the focal physician’s trust after joining a team? In other words, how should the physician choose a team to best improve her trust?

To answer these research questions, this study proposes a research model based on the brand extension theory. Although trust can be provided by the platform design, its impacts are out of the scope of our research purpose. Two team characteristics (team influence and team similarity) are hypothesized to have the main effects. Another team characteristic, the team size, is hypothesized to negatively moderate the impacts of team influence and team similarity on physician trust. A 2 × 2 × 2 experiment was conducted to test the proposed research model. The experiment is an appropriate research methodology for this study because physician trust is a physiological variable that cannot be observed directly from the user’s behavior. Instead, it can only be measured through surveys, using questionnaires. Since we investigated the physician trust on an e-consultation platform, some IT system stimulus is necessary. Therefore, an experiment that can provide such a stimulus for each participant and then measure his/her user perception is an appropriate design for this study. In addition, the experiment that randomly assigns participants to different groups and better controls for other confounding factors provides more effective causal explanations than the survey and the secondary data econometric analysis [23].

The remainder of this paper is organized as follows. Section 2 presents the research model and the hypotheses. Section 3 introduces the research methodology and the data analysis, and the results are then presented in Section 4. In Section 5, the major findings, contributions, and implications of this research are addressed accordingly. Finally, this paper concludes with a discussion on the limitations of the study and directions for future research.

## 2. The Research Model and Hypothesis Development

**Figure 1:**
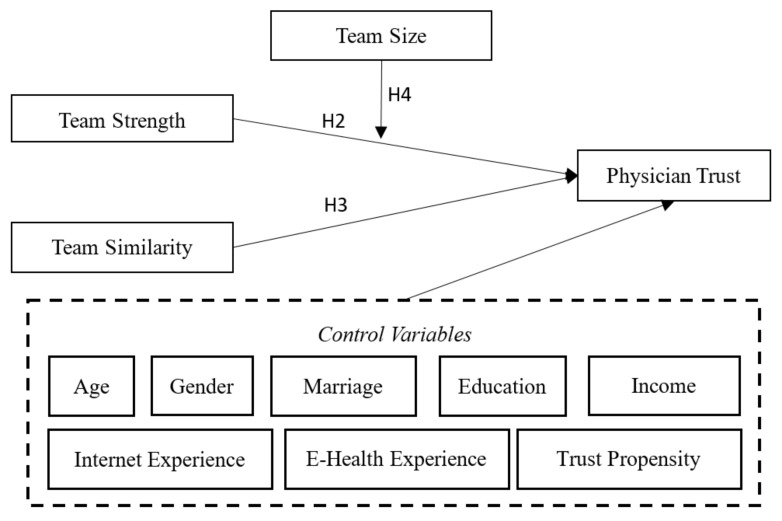
The research model.

To address the limitations of prior research, and answer the proposed research questions, a research model was developed; shown in Figure 1. We identified three team characteristics, namely, team strength, team similarity and team size, which could influence physician trust. Team influence and team similarity were hypothesized to have the main effects, and team size was hypothesized to have a moderating effect. The control variables included age, gender, marriage, education, income, internet experience, e-health experience, and trust propensity. 

There are many trust benefits for the physician who joins a team. First, joining a team means better collaboration among physicians, which is important for modern medicine. Healthcare is now a team effort because modern medicine is becoming more and more complicated [24]. A single physician cannot solve all problems, especially complex problems. A physician team provides an important platform for physicians to discuss illnesses, share experiences, receive latest medical information, reduce errors, and collaborate and address complex situations [9]. Therefore, joining a team provides some advantages for physicians in the delivery of high-quality services. Second, being accepted by a high-quality team is an endorsement of physician quality because only high-quality physicians can join a high-quality team. Therefore, joining a good team sends a message about a physician’s quality [9]. Given this information, we expect that a user will have greater trust in physicians who have joined a team. Therefore, we have proposed the following hypothesis:

**Hipothesis** **1:**  *Users perceive higher trust in physicians who work in a medical team than those who do not.*

Brand extension is a common method used by companies to launch a new product using an existing brand name for the new product in a different category [25]. Companies use brand extension to leverage their existing customer base and brand loyalty to increase profits with a new product offering. The associative network memory model is usually used as the theoretical foundation to explain the effect of brand extension [26]. This model suggests that information is stored in the brain as nodes which are linked to other nodes. A node can activate other nodes through their association. In the brand extension context, each node contains information of a brand or a product. When the brand node is activated, it spreads to other nodes that have been linked to the particular brand. Therefore, when a brand is closely linked with a positive image, even if the extension brand is strange to the customers, it becomes a potential source of activation for the image node [27]. Although the brand extension theory origins from the marketing literature, its application has recently expanded in the online health platform context [27].

The brand extension theory suggest two important sources of a successful brand extension—parent brand strength and parent–extension similarity [25,28]. Parent brand strength is the consumer predispositions toward the parent brand [28]. Brand strength is also known as brand attitude, i.e., the consumer perceptions of quality associated with a brand [29]. The strength of a brand is related to its ability to reduce perceived risk. If a brand is associated with high quality, the extension should benefit; if it associated with inferior quality, the extension should be harmed [29]. Parent–extension similarity is the degree to which consumers perceive the extension as similar to the parent brand [28]. The higher the similarity, the higher perceived risk relief by consumers because the extension from parent to extension is easier to succeed when the similarity is high [25].

In the online physician team context, the parent brand is the physician team. Therefore, we extend parent brand strength as team strength in this research and define it as the perceived quality of the physician team. As suggested by brand extension theory, the parent brand strength is positively related to its ability to reduce perceived risk. Parent brands with higher perceived quality should provide greater risk relief than lower quality brands. If the team members and the team leader are perceived as well-known, highly competent, experienced, friendly or highly rated, then the team strength is also high. We expect that a physician will gain more trust if she joins a team with high strength. Therefore, we have proposed the following hypothesis:

**Hipothesis** **2:**  *Team strength has a positive influence on physician trust.*

In the online physician team context, we extend parent–extension similarity as team similarity and define it as the similarity between the focal physician and other members in the team. The similarity between the focal physician and other team members can be measured by distances from several dimensions, such as technique distance, geographic distance, or grade distance [9]. The higher the distance, the lower the similarity. According to the brand extension theory, consumers’ beliefs about referent brands tend to transfer to an extension most readily, when the extension is perceived as highly related to the corresponding parent brands [28]. In contrast, consumers tend to be skeptical of extensions that they perceive as deviating too far from a company’s historic domain of expertise [29]. If the focal physician is dissimilar to the team members and the team leader, then the quality endorsement from the team will be less reliable. In addition, the collaboration, communication, and experience sharing will be less effective if there are huge distances between the focal physician and the other members of the team. We expect that a physician will gain more trust if she joins a team with high similarity. Therefore, we have proposed the following hypothesis:

**Hipothesis** **3:**  *Team similarity has a positive influence on physician trust.*

Team size is the number of physicians that belongs to a team. Team size is an important variable that predicts the group identification [30,31]. When the team size is small, each member is viewed as important and essential. However, when the team size is large, each member is less important to the team. This means that the endorsement effect of joining a team will decrease as the team size increases. If the team size is very large, each team member will no longer be viewed as elite [32]. Therefore, we expect that the impact of team strength on trust will be weaker when the team size is large. Therefore, we have proposed the following hypothesis:

**Hipothesis** **4:**  *A larger team size corresponds to a lesser positive effect of team strength on physician trust.*

## 3. Research Methodology

In this section, we first discuss our experimental design and then describe the measures for the constructs.

### 3.1. Experimental Design

A 2 (team strength—high vs. low) × 2 (team similarity—high vs. low) × 2 (team size—big vs. small) experiment was conducted to evaluate the proposed research hypotheses. All three factors (i.e., team strength, team similarity, and team size) were between-subjects factors. To test the effect of joining a team on physician trust, another non-team condition was added. Therefore, we had 9 conditions in total. Subjects were shown 2 physicians in a sequential manner. Among the two physicians, one had joined a team and the other had not. For the physician who had joined a team, we randomly showed 1 out of the 8 conditions. 

**Figure 2:**
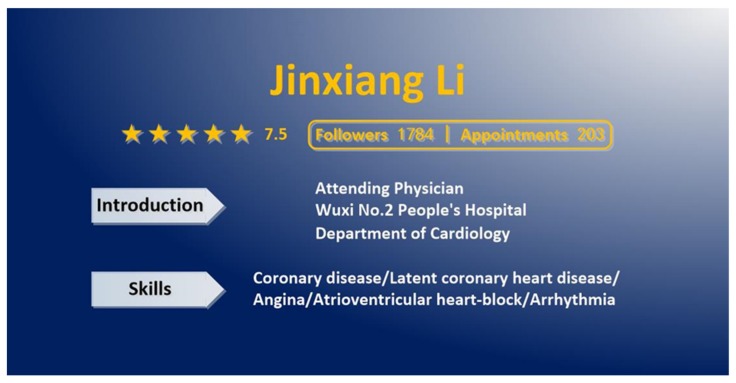
The description of the physician who did not join any team.

We designed 9 fictitious physician descriptions. To ensure that trust was influenced only by the team-related factors, the physician who had joined a team and the physician who had not joined a team were almost identical in terms of personal information such as medical skills, title, rating score, the number of followers and the number of appointments. However, the physicians’ names and the affiliated hospital were different to ensure that a subject felt that she was judging two different physicians. The description for the physician who did not join a team is shown in Figure 2. Only the physician’s personal information such as name, rating score, the number of followers, the number of appointments, a brief introduction, and medical skills are presented.

**Figure 3:**
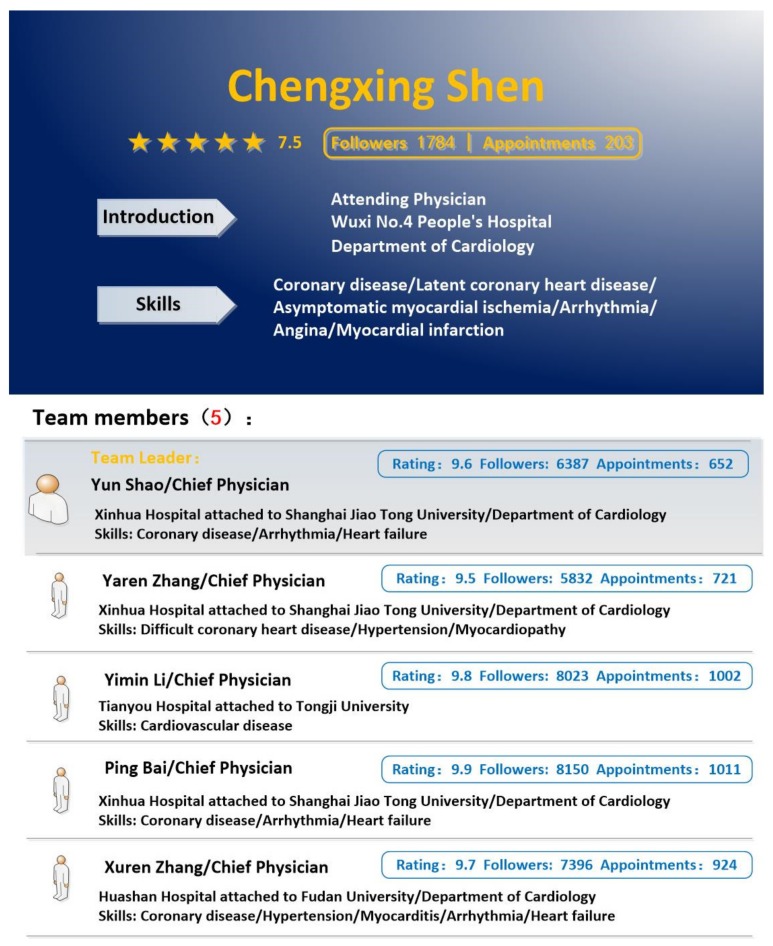
The description of the physician who joined a team.

The description of the physician who joined a team is shown in Figure 3. The description in Figure 3 consists of two parts—personal information and team information. The personal information in Figure 3 is almost identical to Figure 2. The newly added team information included name, title, hospital name, skills, rating score, the number of followers, and the number of appointments for the team leader and the other team members. The team strength was manipulated by the rating score, the number of followers, and the number of appointments. The team similarity was manipulated by the similarity of medical skills, the geographic distance, and the title/position distance. Five members were manipulated as the small team size and 25 members were manipulated as the large team size. 

An online survey was used to carry out the experiment. The subjects were recruited voluntarily from a public forum in China in June 2019. We selected a famous public forum in China (tianya.cn) and posted the online survey invitation link. To better motivate user participation, the subjects who completed a questionnaire received a telecom recharge card of 30 yuan (around 4.3 US dollars). The subjects were asked to provide demographic information first and were then shown 2 physician descriptions (one who had joined a team and one who had not). The subjects were asked to rate physician trust using a 1–7 Likert scale upon seeing the physician description. For the physician who joined a team, the subjects were also asked to rate the perceived team strength, the perceived team similarity, and the perceived team size, also using a 1–7 Likert scale. To minimize the confounding learning effect, the sequence of the two physicians was counterbalanced for each subject. The institutional review board approval was obtained for this study.

We obtained a total of 258 valid questionnaires after removing incomplete cases. Since we randomly assigned 1 of the 8 conditions for each subject, there were approximately 32 samples for each condition. The demographic information of the subjects is presented in Table 1. As shown in Table 1, around 92% of respondents were between 20 and 40 years old. That is because e-consultation is still a new concept in China. Older people lack the ability to use information technology, or are less receptive to new things, which makes them less interested in e-consultation. Therefore, the subjects were a good representation of e-consultation users.

Given that the data were collected from a single source at the same time and were perceptual, we further tested common method bias. We followed Harman’s single-factor method [33] to evaluate the four conceptual variables in our model. The results revealed that all four constructs had eigenvalues greater than 1, and the first factor accounted for 38.65% of the variance. Therefore, the threat of common method bias for the results was not a major issue for this study.

### 3.2. Measures

The measures for the constructs were based on previous studies, with adjustments for the specific research context. All items were measured on a seven-point Likert-type scale with anchors of ‘strongly disagree’ (one) to ‘strongly agree’ (seven). The measures for the constructs are shown in Table 2.

## 4. Data Analysis and Results

For the data analysis, we first analyzed the reliability and two types of validity, i.e., convergent validity and discriminant validity. Then, we checked the impact of joining a team on physician trust using the paired-sample *t*-test. Lastly, we assessed the structural model and tested the hypotheses. In our research, analyses of both a measurement model and a structural model were provided by SPSS (SPSS Inc., Chicago, IL, USA) and SmartPLS (SmartPLS GmbH, Boenningstedt, Germany) [36].

### 4.1. The Measurement Model

Reliability is the consistency of a set of measurements revealing a strong mutual interrelation between two outcomes measured by similar methods of the same construct [36]. To assess the reliability of the constructs, we verified the composite reliability (CR), the average variance extracted (AVE), and Cronbach’s Alpha [37,38,39]. As shown in Table 3, both the CR and Cronbach’s Alpha were well above the suggested cut-off value of 0.70. The AVE was equal to or greater than 0.621, which exceeded the accepted threshold of 0.50, thus, indicating a good construct reliability [37,38,39].

Convergent validity relates to the degree to which a scale measuring the same construct provides the same results [37]. The item loadings were examined to assess the convergent validity of constructs and a value less than 0.7 was considered insufficient [40,41,42]. The item loadings in our research ranged from 0.722 to 0.901, which were higher than 0.70 and, thus, provided support for convergent validity.

Discriminant validity is the extent to which a measurement differs between two different constructs [37,38]. From prior research, discriminant validity is tested by comparing the square root of the AVEs and the correlations of this variable with any other model’s constructs [39,40]. In Table 4, we determined that all square roots of the AVEs were higher than the correlations. Therefore, the discriminant validity was acceptable and our measurement model was verified as reliable.

### 4.2. The Impact of Working in a Team on Physician Trust

We ran the paired-sample *t*-test to determine the impact of working in a team on physician trust. Each subject was asked to evaluate trust on two physicians who were nearly identical in terms of personal information, such as medical skills, title, rating score, the number of followers, and the number of appointments. The only difference was that one physician worked in a team and the other one did not. In addition, the two trust judgments were made by the same subject, so the impact from subject-related factors (e.g., age, gender, experience, the convenience of visiting an offline physician, etc.) could be eliminated. Therefore, we could ensure that the differences in trust were totally from the impact of working in a team.

The results of the paired-sample *t*-test indicated that working in a team had a significant impact on physician trust (*t* = −8.035, *p* < 0.001). Therefore, H1 was supported. As shown in Table 5, the physicians who worked in a team had a trust score of 5.57 on average, but the physicians who did not work in a team only had a trust score of 5.01 on average.

### 4.3. The Structural Model

To examine the impacts of team characteristics (i.e., team strength, team similarity, and team size), we compared the Partial Least Squares (PLS) regression results of three models (as shown in Table 6). In Model 1, only the control variables were included. The results showed that eight factors explained 16.7% of the variance of the dependent variable, while gender, marriage, internet experience, and trust propensity were found to be significant. In Model 2, team strength (β = 0.686, *t* = 13.365, *p* < 0.001) and team similarity (β = 0.178, *t* = 3.050, *p* < 0.001) were further included, and the results showed that their impact on physician trust were significant, lending support to H2 and H3. The inclusion of team strength and team similarity increased the R-square value from 0.167 to 0.657.

**Figure 4:**
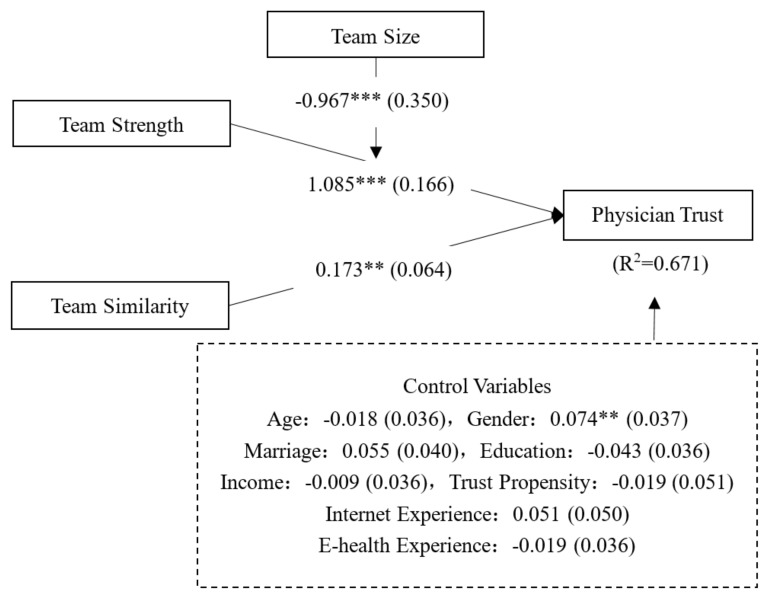
PLS analysis results. *** *p* < 0.001, ** *p* < 0.01, * *p* < 0.05.

In Model 3, the interaction effect of team size and team strength was included. The results showed that the interaction effect was significant (β = –0.967, *t* = 2.590, *p* < 0.001), lending support to H4. The inclusion of the interaction effect increased the R-square value to 0.671. This result suggests that team size and team strength had a significant interaction effect on physician trust. As team size increased, team strength had a less positive effect on physician trust. Figure 4 summarizes the results of the structural model assessment, indicating that all hypotheses (H2–H4) were supported.

## 5. Research Analysis and Discussion

### 5.1. Key Findings

There were several major findings in this study. First, joining a team had a positive effect on physician trust. Due to the transfer of trust from a well-known team leader and other team members, the physicians who joined a team had higher user trust than those who did not. 

Second, both team strength and team similarity had positive impacts on physician trust. Higher team strength corresponded to higher physician trust. In addition, greater similarity between the focal physician and the team members corresponded to higher physician trust.

Third, a larger team size resulted in a reduced positive effect of team strength on physician trust, because as team size increased, the endorsement effect of joining a team decreased. The team member would no longer be viewed as elite when the team size increased beyond a certain number of physicians.

### 5.2. Theoretical Contribution

This study makes several theoretical contributions. First, this was the first empirical study to address the important research question of how to increase the initial trust in an unknown online physician. Specifically, we focused on whether joining a team could increase physician trust and what team characteristics would take effect. Although the collaboration of caregivers and physician trust have been investigated by many researchers, very few studies have addressed the research question of how to increase online physician trust through team organization. 

Second, this study employed a brand extension theory to explain the impact of joining a team on physician trust. Although the brand extension theory has been widely used in marketing literature, its application in healthcare remains insufficient. In this study, we extended two important concepts of brand extension theory to the healthcare context. Parent-brand strength was extended as team strength, and the similarity between a parent brand and an extension product was extended as team similarity. Our results indicated that the extensions were successful.

### 5.3. Implications

The findings of the current study had several practical implications. 

First, joining a team is an effective and low-cost method to increase online physician trust. This finding is significant for both the unknown physicians and the e-consultation website. For an unknown physician, the findings of this study indicate that joining a team could increase user trust, which is an important antecedent of online transaction intention. Therefore, we can expect that a physician who joined a team will have a higher chance of obtaining more online orders and will also acquire a higher online income. For the e-consultation website, the study findings indicate that a physician team is an effective and low-cost method to increase a user’s trust in the physicians on the platform. The physician team appears to be a promising solution to alleviate the initial trust problem.

Second, the results of this study indicate that a physician should join a team that is high in strength or highly similar to herself. Higher team strength corresponds to higher similarity between the focal physician and the team members and greater trust gained. In addition, given the same level of team strength, a physician should choose a small team. Therefore, team strength, team similarity and team size are all important variables that a physician must consider when she makes the decision to join a team.

## 6. Limitations and Future Research

There are several limitations of this research. First, all variables used in this study contained only self-reported data. Future studies should include the actual transaction data (rather than the trust intention in this study) to increase research validity.

Second, this study only investigated the impact of three team characteristics (i.e., team strength, team similarity, and team size) on physician trust. The other variables identified by the brand extension theory (e.g., parent-brand experience, marketing support, history of previous brand extensions, consumer knowledge, and consumer innovativeness) might also have impacts on physician trust. Therefore, future studies could benefit from investigating the effects of these variables.

This study has significant implications for both unknown physicians and e-consultation websites. However, the effect of joining a physician team was only validated in the experiment context. Therefore, the generalizability of the research findings needs to be strengthened. In future research, we intend to investigate its effect in real websites, and a field experiment or a quasi-field experiment is highly desirable to test its effect in the real settings. 

## Figures and Tables

**Table 1:** Subject demographics.

Variables	Values	Frequency	Percentage
Gender	Male	112	43.41%
	Female	146	56.59%
Age (years)	20–30	102	39.53%
	30–40	137	53.10%
	Others	19	7.36%
Education	Senior high school or less	8	3.10%
	Junior college	26	10.08%
	Undergraduate	211	81.78%
	Postgraduate	13	5.04%
Income(Chinese Yuan)	<1000	2	0.78%
	1000–3000	17	6.59%
	3001–5000	54	20.93%
	5001–8000	110	42.64%
	8001–15000	61	23.64%
	>15000	14	5.43%

**Table 2:** Constructs and items.

Constructs	Items		Literature
Team Strength	TS1	The team members are high in medical competence.	Self-developed
	TS2	The team members are high in reputation.	
	TS3	The team leader is well-known and prestigious.	
	TS4	In general, the medical team is high in strength.	
Team Similarity	TSM1	The focal physician is similar to the team members in terms of medical skills.	Self-developed
	TSM2	The focal physician is geographically near the team members.	
	TSM3	The focal physician is similar to the team members in terms of title or position.	
	TSM4	In general, the focal physician is similar to the team members.	
Trust Propensity	TP1	It is easy for me to trust a person/thing.	Adapted from [34]
	TP2	My tendency to trust a person/thing is high.	
	TP3	I tend to trust a person/thing, even though I have little knowledge of it.	
	TP4	Trusting someone or something is not difficult.	
Physician Trust	PT1	This physician is trustworthy.	Adapted from [34,35]
	PT2	This physician is qualified to me.	
	PT3	I believe in the information that this physician provides me.	
	PT4	I will follow the physician’s advice.	
Team Size	TSI1	The team size is large.	Self-developed
Internet Experience	IE1	I have rich experience in using the Internet.	Self-developed
E-health Experience	EE1	I have rich experience in using online health services.	Self-developed

**Table 3:** Reliability and convergent validity.

Construct	CR	AVE	Cronbach’s Alpha	Item	Loading	T-Statistics
Team Strength	0.883	0.654	0.823	TS1	0.866	50.794
				TS2	0.803	28.984
				TS3	0.739	16.655
				TS4	0.823	30.429
Team Similarity	0.867	0.621	0.803	TSM1	0.722	13.258
				TSM2	0.758	18.247
				TSM3	0.835	32.928
				TSM4	0.832	28.691
Trust Propensity	0.919	0.740	0.884	TP1	0.883	40.376
				TP2	0.880	34.316
				TP3	0.779	17.312
				TP4	0.894	50.028
Physician Trust	0.928	0.764	0.897	PT1	0.838	32.209
				PT1	0.880	45.477
				PT3	0.876	42.035
				PT4	0.901	51.329

Note: CR = Composite Reliability, AVE = Average Variance Extracted.

**Table 4:** Discriminant validity.

Construct	Team Strength	Team Similarity	Trust Propensity	Physician Trust
Team Strength	**0.809**			
Team Similarity	0.364	**0.788**		
Trust Propensity	0.271	0.192	**0.860**	
Physician Trust	0.782	0.451	0.272	**0.874**

Note: The bold diagonal data refer to the square roots of the AVEs.

**Table 5:** Trust in the physician who worked in a team and the one who did not.

Condition	Mean	Std. Dev.	Sample Size
Worked a team	5.57	0.982	258
Did not work a team	5.01	1.084	258

**Table 6:** Partial Least Squares (PLS) results.

Variables	Model 1	Model 2	Model 3
Age	−0.006	−0.021	−0.018
	(0.077)	(0.034)	(0.036)
	*t* = 0.078	*t* = 0.615	*t* = 0.496
Gender	0.150 ***	0.069 *	0.074 **
	(0.058)	(0.037)	(0.037)
	*t* = 2.758	*t* = 1.925	*t* = 2.051
Marriage	0.115 *	0.068 *	0.055
	(0.060)	(0.043)	(0.040)
	*t* = 1.859	*t* = 1.737	*t* = 1.335
Education	−0.023	−0.043	−0.043
	(0.061)	(0.037)	(0.036)
	*t* = 0.403	*t* = 1.132	*t* = 1.168
Income	−0.031	−0.008	−0.009
	(0.068)	(0.037)	(0.036)
	*t* = 0.496	*t* = 0.225	*t* = 0.248
Internet Experience	0.220 ***	0.044	0.051
	(0.086)	(0.050)	(0.050)
	*t* = 2.733	*t* = 0.869	*t* = 1.041
E-health Experience	−0.001	−0.019	−0.019
	(0.072)	(0.037)	(0.036)
	*t* = 0.017	*t* = 0.524	*t* = 0.502
Trust Propensity	0.235***	0.042	−0.019
	(0.068)	(0.056)	(0.051)
	*t* = 3.327	*t* = 0.742	*t* = 0.731
Team Strength		0.686 ***	1.085 ***
		(0.052)	(0.166)
		*t* = 13.365	*t* = 6.182
Team Similarity		0.178 ***	0.173 **
		(0.060)	(0.064)
		*t* = 3.050	2.523
Team Size			0.749 **
			(0.290)
			*t* = 2.428
Team Size *Team Strength			−0.967 ***
			(0.350)
			*t* = 2.590
Observations	258	258	258
$R^{2}$	0.167	0.657	0.671

Note: *** *p* < 0.001, ** *p* < 0.01, * *p* < 0.05.
